# The Advisory Committee on Immunization Practices’ Interim Recommendation for Use of Moderna COVID-19 Vaccine — United States, December 2020

**DOI:** 10.15585/mmwr.mm695152e1

**Published:** 2021-01-01

**Authors:** Sara E. Oliver, Julia W. Gargano, Mona Marin, Megan Wallace, Kathryn G. Curran, Mary Chamberland, Nancy McClung, Doug Campos-Outcalt, Rebecca L. Morgan, Sarah Mbaeyi, José R. Romero, H. Keipp Talbot, Grace M. Lee, Beth P. Bell, Kathleen Dooling

**Affiliations:** ^1^CDC COVID-19 Response Team; ^2^Epidemic Intelligence Service, CDC; ^3^General Dynamics Information Technology, Falls Church, Virginia; ^4^University of Arizona, College of Medicine, Phoenix, Arizona; ^5^Department of Health Research Methods, Evidence, and Impact, Hamilton, Ontario, Canada; ^6^Arkansas Department of Health; ^7^Vanderbilt University School of Medicine, Nashville, Tennessee; ^8^Stanford University School of Medicine, Stanford, California; ^9^University of Washington, Seattle, Washington.

On December 18, 2020, the Food and Drug Administration (FDA) issued an Emergency Use Authorization (EUA) for the Moderna COVID-19 (mRNA-1273) vaccine (ModernaTX, Inc; Cambridge, Massachusetts), a lipid nanoparticle-encapsulated, nucleoside-modified mRNA vaccine encoding the stabilized prefusion spike glycoprotein of SARS-CoV-2, the virus that causes coronavirus disease 2019 (COVID-19) (*1*). This vaccine is the second COVID-19 vaccine authorized under an EUA for the prevention of COVID-19 in the United States (*2*). Vaccination with the Moderna COVID-19 vaccine consists of 2 doses (100 *μ*g, 0.5 mL each) administered intramuscularly, 1 month (4 weeks) apart. On December 19, 2020, the Advisory Committee on Immunization Practices (ACIP) issued an interim recommendation* for use of the Moderna COVID-19 vaccine in persons aged ≥18 years for the prevention of COVID-19. To guide its deliberations regarding the vaccine, ACIP employed the Evidence to Recommendation (EtR) Framework,^†^ using the Grading of Recommendations Assessment, Development and Evaluation (GRADE) approach.^§^ Use of all COVID-19 vaccines authorized under an EUA, including the Moderna COVID-19 vaccine, should be implemented in conjunction with ACIP’s interim recommendations for allocating initial supplies of COVID-19 vaccines (*3*). The ACIP recommendation for the use of the Moderna COVID-19 vaccine under EUA is interim and will be updated as additional information becomes available.

Since June 2020, ACIP has convened 10 public meetings to review data on the epidemiology of COVID-19 and the potential use of COVID-19 vaccines, including the Moderna COVID-19 vaccine (*4*). Within the EtR Framework, ACIP considered the importance of the public health problem of COVID-19, as well as resource use, benefits and harms, patients’ values and preferences, acceptability, feasibility, and equity for the Moderna COVID-19 vaccine. To inform the EtR Framework, the COVID-19 Vaccines Work Group, comprising experts in infectious diseases, vaccinology, vaccine safety, public health, and ethics, held 28 meetings to review COVID-19 surveillance data, evidence for vaccine efficacy and safety, and implementation considerations for COVID-19 vaccines, including the Moderna COVID-19 vaccine. After a systematic review of available data, the Work Group used the GRADE approach to assess the certainty of evidence for outcomes related to the vaccine, rated on a scale of 1 (high certainty) to 4 (very low certainty) (*5*). Work Group conclusions regarding certainty of evidence for the Moderna COVID-19 vaccine were presented to ACIP at public meetings.

The body of evidence for the Moderna COVID-19 vaccine was primarily informed by one large, randomized, double-blind, placebo-controlled Phase III clinical trial that enrolled approximately 30,000 participants aged 18–95 years (median = 52 years) (*6**–**9*). Interim findings from this clinical trial, using data from participants with a median of 2 months of follow-up, indicate that the Moderna COVID-19 vaccine efficacy after 2 doses was 94.1% (95% confidence interval = 89.3%–96.8%) in preventing symptomatic, laboratory-confirmed COVID-19 among persons without evidence of previous SARS-CoV-2 infection, which was the primary study endpoint. High efficacy (≥86%) was observed across age, sex, race, and ethnicity categories and among persons with underlying medical conditions. Ten hospitalizations due to COVID-19 were documented; nine in the placebo group and one in the vaccine group (*9*). Preliminary data suggest that the Moderna COVID-19 vaccine might also provide some protection against asymptomatic SARS-CoV-2 infection (*7*). Among vaccine recipients, reactogenicity symptoms, defined as solicited local injection site or systemic adverse reactions during the 7 days after vaccination, were frequent but mostly mild to moderate. Systemic adverse reactions were more commonly reported after the second dose than after the first dose and were more frequent and severe in persons aged 18–64 years than in those aged ≥65 years. Most local and systemic adverse reactions occurred within the first 1–2 days after vaccine receipt and resolved in a median of 2–3 days. Severe local or systemic adverse reactions (grade ≥3 reactions^¶^) occurred more commonly in vaccine recipients than in placebo recipients (21.6% versus 4.4%). Among vaccine recipients, 9.1% reported a grade ≥3 local injection site reaction, and 16.5% reported a grade ≥3 systemic adverse reaction. The frequency of serious adverse events** observed was low in both the vaccine (1.0%) and placebo (1.0%) recipients and without meaningful imbalances for specific serious adverse events between the two groups (*8*). No specific safety concerns were identified in subgroup analyses by age, race, ethnicity, underlying medical conditions, or previous SARS-CoV-2 infection. A detailed summary of safety data, including information on reactogenicity, is available at https://www.cdc.gov/vaccines/covid-19/info-by-product/moderna/reactogenicity.html.

From the GRADE evidence assessment, the level of certainty for the benefits of the Moderna COVID-19 vaccine was type 1 (high certainty) for the prevention of symptomatic COVID-19. Evidence was type 2 (moderate certainty) for the estimate of prevention of COVID-19–associated hospitalization and type 4 (very low certainty) for the estimates of prevention of asymptomatic SARS-CoV-2 infection and all-cause death. Data on COVID-19–associated hospitalizations and deaths are limited at this time; however, a vaccine that effectively prevents symptomatic infection is expected to also prevent associated hospitalizations and deaths. Regarding certainty of evidence related to possible harms after vaccination, evidence was type 2 (moderate certainty) for the estimate of serious adverse events and type 1 (high certainty) for the estimate of reactogenicity. Data reviewed within the EtR Framework supported the use of the Moderna COVID-19 vaccine. ACIP determined that COVID-19 is a major public health problem and that use of the Moderna COVID-19 vaccine is a reasonable and efficient allocation of resources. Whereas there might be uncertainty about how all populations value the vaccine, it was determined that for most populations, the desirable effects outweigh the undesirable effects, making the vaccine acceptable to implementation stakeholders. In addition, implementation of administration of the Moderna COVID-19 vaccine is feasible. Although the vaccine requires a freezer (–20^o^C [–4^o^F]) for long-term storage, it is stable at refrigerator temperatures (2–8^o^C [35–46^o^F]) for up to 30 days after thawing. This characteristic will facilitate feasibility of administration of the Moderna COVID-19 vaccine in most community settings, once supply allows. Advancing health equity, however, will require efforts to identify and reduce access-related barriers to vaccination, as well as engagement with community organizations and leaders among groups who experience disproportionate COVID-19–related morbidity and mortality, and to expand access to clear and accurate information on COVID-19 vaccines (*10*). The GRADE evidence profile and supporting evidence for the EtR Framework are available at https://www.cdc.gov/vaccines/acip/recs/grade/covid-19-moderna-vaccine.html and https://www.cdc.gov/vaccines/acip/recs/grade/covid-19-moderna-etr.html.

Before vaccination, the EUA Fact Sheet (*11*) should be provided to recipients and caregivers. Providers should counsel Moderna COVID-19 vaccine recipients about expected local and systemic reactogenicity. The Moderna COVID-19 vaccine is not interchangeable with other COVID-19 vaccine products; the safety and efficacy of a mixed-product series have not been evaluated. ACIP does not state a product preference; a person may receive any recommended COVID-19 vaccine series. However, persons should complete the series with the same COVID-19 product they received for the first dose. Additional clinical considerations, including details of administration and use in special populations (e.g., persons who are pregnant, immunocompromised or who have a history of severe allergic reactions) are available at https://www.cdc.gov/vaccines/covid-19/info-by-product/clinical-considerations.html. The interim recommendation and clinical considerations are based on use of the Moderna COVID-19 vaccine under an EUA and might change as more evidence becomes available. ACIP will continue to review additional data as they become available; updates to recommendations or clinical considerations will be posted on the ACIP website (*3*).

## Reporting of Vaccine Adverse Events

Adverse events that occur in a recipient after receipt of COVID-19 vaccine should be reported to the Vaccine Adverse Events Reporting System (VAERS). FDA requires that vaccination providers report vaccination administration errors, serious adverse events, cases of multisystem inflammatory syndrome, and cases of COVID-19 that result in hospitalization or death after administration of COVID-19 vaccine under EUA. Reporting by anyone who gives or receives a COVID-19 vaccine is encouraged for any clinically significant adverse event, whether or not it is clear that a vaccine caused the adverse event. Information on how to submit a report to VAERS is available at https://vaers.hhs.gov/index.html or 1-800-822-7967. In addition, CDC has developed a new, voluntary smartphone-based tool, v-safe, that uses text messaging and web surveys to provide near real-time health check-ins after patients receive COVID-19 vaccination. The CDC/v-safe call center follows up on reports to v-safe that indicate a medically significant health impact to collect additional information for completion of a VAERS report. Information on v-safe is available at https://www.cdc.gov/vsafe. Information on how to use both reporting systems is included in the EUA Fact Sheet (*11*).

**Summary**

**What is already known about this topic?**

On December 18, 2020, the Food and Drug Administration issued an Emergency Use Authorization (EUA) for the Moderna COVID-19 vaccine.

**What is added by this report?**

On December 19, 2020, after a transparent, evidence-based review of available data, the Advisory Committee on Immunization Practices (ACIP) issued an interim recommendation for use of the Moderna COVID-19 vaccine in persons aged ≥18 years for the prevention of COVID-19.

**What are the implications for public health practice?**

Use of all COVID-19 vaccines authorized under an EUA, including the Moderna COVID-19 vaccine, should be implemented in conjunction with ACIP’s interim recommendations for allocating initial supplies of COVID-19 vaccines.

## Footnotes

* On December 19, 2020, ACIP voted 11–0 in favor of the interim recommendation for use of the Moderna COVID-19 vaccine. Three ACIP members recused themselves because of participation in clinical trials and/or other studies involving companies producing COVID-19 vaccines.

† https://www.cdc.gov/vaccines/acip/recs/grade/downloads/ACIP-evidence-rec-frame-508.pdf.

§ https://www.cdc.gov/vaccines/acip/recs/grade/about-grade.html.

¶ Grade 3 reactions are defined as use of a prescription pain reliever or those preventing daily activity, fever (temperature 102.1–104.0^o^F [39–40^o^C]); grade 4 reactions are defined as those that require emergency department visit or hospitalization, temperature >104^o^F (40^o^C).

** Serious adverse events are defined as any untoward medical occurrence that results in death, is life-threatening, requires inpatient hospitalization or prolongation of existing hospitalization, or results in persistent disability/incapacity.
